# Perioperative Factors Associated with 48-Hour Postoperative Oxycodone Requirement After Gastrointestinal Surgery: A Single-Center Retrospective Cohort Study

**DOI:** 10.3390/jcm15155957

**Published:** 2026-07-30

**Authors:** Yu-qian Chen, Dan-dan Chen, Xiao-quan Yu, Yi-xian Li, Shun-yuan Li

**Affiliations:** Department of Anesthesiology, Quanzhou First Hospital, Quanzhou 362000, China

**Keywords:** gastrointestinal surgery, oxycodone, patient-controlled analgesia, postoperative pain, perioperative analgesia, opioid requirement, retrospective cohort study

## Abstract

**Background/Objectives:** Postoperative opioid requirements vary substantially after gastrointestinal surgery. Oxycodone-based patient-controlled intravenous analgesia (PCIA) is used for postoperative pain control, but perioperative factors associated with early postoperative oxycodone requirement remain insufficiently defined. This study investigated factors associated with 48 h oxycodone consumption after gastrointestinal surgery. **Methods:** This single-center retrospective cohort study included 493 adults who underwent elective gastrointestinal surgery and received oxycodone-based PCIA between January and December 2025. The primary outcome was cumulative oxycodone consumption within 48 h after surgery. Multivariable linear regression was used for the primary continuous-outcome analysis. An exploratory analgesia-adjusted model additionally included regional block, incisional local infiltration, and routine postoperative non-opioid analgesic use. High consumption, defined as a 48 h dose >38 mg, was evaluated using exploratory logistic regression models. **Results:** The median 48 h oxycodone consumption was 34 mg (interquartile range, 27–38 mg), and 105 patients (21.3%) had high consumption. In the prespecified clinical continuous-outcome model, gastric surgery, rectal surgery, open surgery, converted-to-open surgery, radical resection, and longer operation time were associated with higher 48 h oxycodone consumption, whereas older age was associated with lower consumption. After adjustment for available analgesia-related variables, the main associations remained broadly stable, although the association for converted-to-open surgery was attenuated. Regional block and incisional local infiltration were associated with lower 48 h oxycodone consumption in the analgesia-adjusted model. In the exploratory logistic models, younger age, rectal surgery, open or converted-to-open surgery, radical resection, and longer operation time were associated with high consumption. Body mass index (BMI) was not independently associated with oxycodone requirement. **Conclusions:** Age and procedure-related characteristics were associated with 48 h oxycodone requirement after gastrointestinal surgery. These routinely available perioperative factors may help identify patients who warrant earlier postoperative pain reassessment, closer acute pain service follow-up, and individualized multimodal analgesic planning. Prospective multicenter validation is required before formal risk-stratified analgesic protocols can be recommended.

## 1. Introduction

Acute postoperative pain remains common after gastrointestinal surgery and may delay early mobilization, impair respiratory and gastrointestinal recovery, reduce sleep quality, and prolong postoperative rehabilitation [1,2]. Patient-controlled intravenous analgesia (PCIA) is widely used for individualized postoperative opioid titration, particularly after abdominal procedures involving both visceral and somatic pain [2,3]. However, even among patients receiving similar PCIA regimens, actual postoperative opioid consumption can vary substantially. This variability may reflect patient characteristics, surgical trauma, anesthetic and analgesic exposure, postoperative pain intensity, and opioid-related adverse effects or subsequent analgesic adjustments [1,2,3].

Oxycodone is a semi-synthetic opioid that has been used for the treatment of moderate to severe postoperative pain [4]. Because postoperative pain after gastrointestinal surgery often includes a visceral component, oxycodone may be clinically relevant in this setting [4]. Evidence from systematic reviews and meta-analyses suggests that intravenous oxycodone can provide effective postoperative analgesia, including in PCIA settings, although its efficacy and adverse-effect profile depend on dose, regimen, and surgical population [5,6]. A randomized trial in elderly patients undergoing laparoscopic surgery for gastrointestinal cancer showed that oxycodone PCIA provided postoperative analgesia, with bolus dose selection affecting analgesic efficacy, PCIA demands, cumulative consumption, dizziness, and patient satisfaction [7]. Nevertheless, that trial focused on optimal bolus dose selection in an elderly laparoscopic cancer population, whereas real-world perioperative factors associated with high postoperative oxycodone requirements across broader gastrointestinal surgical procedures remain insufficiently defined.

Previous retrospective PCIA studies have used the third quartile (Q3) of 48 h opioid consumption to define a high-consumption group, providing a pragmatic approach to identifying patients with relatively high postoperative opioid requirements. In patients undergoing hepatectomy, younger age, major resection, open surgery, and longer operation time were independently associated with higher 48 h sufentanil consumption [3]. Similarly, a retrospective study of hydromorphone PCIA after gynecologic surgery reported that age, height, surgical approach, resection extent, operation time, and postoperative nausea and vomiting were associated with 48 h hydromorphone consumption [8]. However, whether these factors also apply to oxycodone-based PCIA after gastrointestinal surgery remains insufficiently defined.

Evidence specific to 48 h oxycodone consumption during PCIA after gastrointestinal surgery remains limited. Identifying patients at risk of high postoperative oxycodone requirement may help clinicians refine preoperative counseling, perioperative analgesic planning, postoperative acute pain service (APS) follow-up, and individualized multimodal analgesic strategies. Therefore, this study aimed to describe the distribution of 48 h oxycodone consumption in patients receiving oxycodone-based PCIA after gastrointestinal surgery and to identify perioperative factors associated with 48 h postoperative oxycodone requirement. High 48 h oxycodone consumption was further examined as an exploratory binary outcome.

## 2. Materials and Methods

### 2.1. Study Design and Setting

This single-center retrospective cohort study was conducted at Quanzhou First Hospital, Quanzhou, Fujian, China. We reviewed routinely collected perioperative records, anesthesia records, postoperative analgesia records, and acute pain service (APS) follow-up records of adult patients who underwent gastrointestinal surgery and received oxycodone-based patient-controlled intravenous analgesia (PCIA). The retrospective data extraction period covered surgeries performed between 1 January 2025 and 31 December 2025, as specified in the approved retrospective study protocol.

The study was based entirely on existing clinical data and did not alter clinical care, anesthetic management, medication selection, postoperative analgesia, or follow-up pathways. The final locked dataset included 493 eligible patients. The study was designed and reported in accordance with the Strengthening the Reporting of Observational Studies in Epidemiology (STROBE) statement where applicable [9].

The study was approved by the Ethics Committee of Quanzhou First Hospital (approval number: QYLL-2026-K145). The requirement for written informed consent was waived because of the retrospective nature of the study and the use of de-identified clinical data. The study was retrospectively registered in the Chinese Clinical Trial Registry (ChiCTR2600123431; registered on 27 April 2026).

### 2.2. Participants

Patients were eligible for inclusion if they were aged 18 years or older, underwent elective gastrointestinal surgery involving the stomach, colon, rectum, or small intestine, received postoperative oxycodone-based PCIA, had an American Society of Anesthesiologists (ASA) physical status of I–III, and had sufficient perioperative and postoperative analgesia records for analysis.

Patients were excluded if they had preoperative chronic pain, long-term regular opioid use or known opioid tolerance, severe psychiatric disease or cognitive impairment that could interfere with pain assessment, combined postoperative strong-opioid analgesic regimens that precluded attribution of oxycodone consumption, key missing variables, reoperation within 48 h after surgery, or major postoperative complications that substantially interfered with postoperative analgesic requirements. Patients were also excluded if 48 h oxycodone consumption could not be reliably obtained or calculated from PCIA records.

Patients with early PCIA discontinuation were retained if the cumulative oxycodone dose within the observation period could be reliably obtained from pump records or medical records. The present analysis used the investigator-confirmed final locked dataset of 493 included patients.

### 2.3. Anesthesia and Postoperative Analgesia

All patients underwent surgery under general anesthesia with endotracheal intubation. Anesthetic management followed institutional perioperative practice but was not mandated by a study protocol. The institutional default induction regimen included intravenous sufentanil 0.5 μg/kg, propofol 2 mg/kg, and cisatracurium 0.2 mg/kg. Anesthesia was maintained using target-controlled infusion of propofol and remifentanil, with dosing adjusted by the attending anesthesiologist according to patient characteristics, surgical requirements, hemodynamic responses, and intraoperative clinical judgment. Additional cisatracurium was administered as needed to maintain neuromuscular blockade. Dolasetron was routinely administered before the end of surgery for prophylaxis against postoperative nausea and vomiting (PONV). Detailed cumulative intraoperative opioid exposure, including total remifentanil administration and any supplemental opioid doses, was not consistently retrievable for the full cohort and was therefore not included in the multivariable models.

Regional block, incisional local infiltration, and routine postoperative non-opioid analgesia were used according to institutional practice and individualized clinical decision-making. These analgesia-related variables were extracted from anesthesia records, surgical records, medication records, and APS follow-up records when documented. Because this was a retrospective real-world study rather than a protocolized interventional study, perioperative analgesic techniques were not assigned by study investigators. Flurbiprofen axetil was the principal non-opioid analgesic used in this cohort; however, the structured retrospective database reliably captured only whether postoperative non-opioid analgesic treatment was documented. Agent-specific information, dose, administration timing, and frequency were not consistently retrievable for all patients. Therefore, routine postoperative non-opioid analgesic use was recorded and analyzed as a binary variable (yes/no).

At the end of surgery, patients were extubated after adequate recovery of consciousness, spontaneous ventilation, and protective airway reflexes, and were transferred to the post-anesthesia care unit (PACU). Patients were subsequently transferred to the ward when PACU discharge criteria were met.

The standard institutional PCIA regimen consisted of oxycodone 50 mg plus dolasetron 25 mg diluted with 0.9% sodium chloride solution to a total volume of 100 mL. Unless clinically adjusted, the pump was programmed to deliver a background infusion of 0.5 mL/h, corresponding to oxycodone 0.25 mg/h. The bolus dose was 2 mL, corresponding to oxycodone 1 mg, with a lockout interval of 10 min. Patients received preoperative instruction on PCIA use. Postoperative pain intensity and opioid-related adverse events were assessed by the ward team and APS team according to routine clinical practice. Rescue analgesia, PCIA parameter adjustment, early PCIA pump discontinuation, and adverse events were recorded when they occurred.

### 2.4. Data Collection and Variables

Data were extracted from electronic medical records, the anesthesia information system, nursing records, APS follow-up records, and PCIA pump or analgesia record forms. Demographic and baseline clinical variables included age, sex, height, body weight, body mass index (BMI), ASA physical status, smoking history, alcohol use, hypertension, and diabetes mellitus. BMI was recalculated as body weight in kilograms divided by height in meters squared.

Surgical variables included disease nature, surgical site, surgical approach, extent of resection, stoma creation, operation time, intraoperative blood loss, and transfusion. Surgical site was categorized as stomach, colon, rectum, or small intestine. Surgical approach was categorized as laparoscopic, open, or converted-to-open surgery. Extent of resection was categorized as local resection or radical resection.

Perioperative analgesia-related variables included regional block, incisional local infiltration, and routine postoperative non-opioid analgesic use. Because agent-specific information, dose, administration timing, and frequency were not consistently available across the full cohort, postoperative non-opioid analgesic use was analyzed as a binary variable (yes/no). Postoperative analgesia and outcome variables included 24 h and 48 h oxycodone consumption, effective and total PCIA demands within 48 h, PCIA parameter adjustment, early PCIA pump discontinuation, numerical rating scale (NRS) pain scores at rest and on movement on postoperative day 1 (POD1) and postoperative day 2 (POD2), PONV within 48 h, rescue analgesia, number of rescue analgesic interventions, opioid-related adverse events, time to first flatus, and postoperative length of stay. Opioid-related adverse events included dizziness, drowsiness or sedation, pruritus, and urinary retention.

### 2.5. Outcomes

The primary outcome was cumulative oxycodone consumption within the first 48 h after surgery, analyzed as a continuous variable. When available, 48 h oxycodone consumption was obtained directly from PCIA pump records or analgesia record forms. If the cumulative dose was not directly recorded, it was calculated according to the recorded PCIA settings, background infusion duration, and number of effective PCIA demands. Under the standard regimen, the background infusion delivered oxycodone 0.25 mg/h, and each effective PCIA demand delivered oxycodone 1 mg.

High 48 h oxycodone consumption was analyzed as an exploratory binary outcome. The third quartile (Q3) of 48 h oxycodone consumption in the final dataset was 38 mg; therefore, high consumption was defined as a 48 h oxycodone dose >38 mg. Patients with 48 h oxycodone consumption ≤38 mg were assigned to the non-high-consumption group. The threshold was used to identify patients with relatively high opioid requirements within this cohort and was not intended to define a universal clinical cutoff.

Secondary outcomes included 24 h oxycodone consumption, postoperative pain scores, PONV, rescue analgesia, opioid-related adverse events, time to first flatus, and postoperative length of stay.

### 2.6. Sample Size and Model Adequacy

Because this was a retrospective cohort study, no formal a priori sample size calculation was performed. All eligible patients who met the inclusion and exclusion criteria during the predefined study period were included. The sample size was therefore determined by the number of eligible patients with complete and reliable PCIA consumption records during the study period.

To reduce the risk of overfitting and data-driven model selection, covariates for the multivariable models were selected a priori based on clinical relevance and availability in routine perioperative records. The primary multivariable analysis treated 48 h oxycodone consumption as a continuous outcome to preserve information and avoid unnecessary loss of statistical power from dichotomization. The exploratory binary analysis of high oxycodone consumption included 105 high-consumption events; therefore, the multivariable logistic regression model was restricted to a prespecified set of clinically relevant preoperative and intraoperative variables. Automated stepwise selection was not used.

### 2.7. Statistical Analysis

Continuous variables were assessed for distributional characteristics and are presented as medians with interquartile ranges (IQRs). Categorical variables are presented as counts and percentages. For descriptive comparisons between patients with and without high 48 h oxycodone consumption, continuous variables were compared using the Mann–Whitney U test, and categorical variables were compared using the chi-square test or Fisher’s exact test, as appropriate. Spearman correlation analysis was used to evaluate associations between continuous or ordinal perioperative variables and cumulative 48 h oxycodone consumption. Between-group comparisons, correlation analyses, and univariable logistic regression analyses were descriptive and exploratory. No adjustment for multiple comparisons was performed, and these analyses were not used for data-driven covariate selection.

The primary outcome was cumulative oxycodone consumption within 48 h after surgery, analyzed as a continuous variable. The primary multivariable analysis evaluated factors associated with 48 h oxycodone consumption using linear regression with heteroscedasticity-robust standard errors. Covariates were selected a priori based on clinical relevance and availability in routine perioperative records, and included age, sex, body mass index (BMI), American Society of Anesthesiologists (ASA) physical status, surgical site, surgical approach, extent of resection, and operation time. Age was entered per 10-year increase, BMI per 1 kg/m^2^ increase, and operation time per 60 min increase. Regression coefficients are reported as adjusted differences in 48 h oxycodone consumption in milligrams, with 95% confidence intervals (CIs).

To address available analgesia-related confounding, an exploratory analgesia-adjusted model was constructed by adding regional block, incisional local infiltration, and routine postoperative non-opioid analgesic use to the prespecified clinical model. This analysis was considered exploratory because these analgesic techniques were not randomly assigned and may have reflected surgical complexity, institutional practice, clinician preference, or patient-specific clinical judgment.

High 48 h oxycodone consumption was analyzed as an exploratory binary outcome. The third quartile of 48 h oxycodone consumption in the final dataset was 38 mg; therefore, high consumption was defined as a 48 h oxycodone dose >38 mg. Univariable logistic regression was first performed to explore variables associated with high consumption. The exploratory multivariable logistic regression model included the same prespecified clinical covariates as the primary continuous-outcome model. An additional exploratory analgesia-adjusted logistic model was also fitted by adding regional block, incisional local infiltration, and routine postoperative non-opioid analgesic use. Results are reported as odds ratios (ORs) or adjusted odds ratios (aORs) with 95% CIs.

Model discrimination for the exploratory logistic models was assessed using the area under the receiver operating characteristic curve (AUC). The 95% CI for the AUC was estimated using bootstrap resampling with 1000 stratified replicates. These logistic models were used to explore factors associated with relatively high oxycodone requirement within this cohort and were not intended to develop or validate a clinical prediction model.

Postoperative pain scores, rescue analgesia, postoperative nausea and vomiting, PCIA parameter adjustment, early PCIA pump discontinuation, and opioid-related adverse events were not included in the main multivariable models because they occurred after surgery or after PCIA initiation and could represent intermediate variables, markers of inadequate analgesia, or consequences of opioid exposure rather than baseline predictors.

Multicollinearity among variables included in the multivariable models was assessed using variance inflation factors. Available-case analysis was used for descriptive and correlation analyses, whereas complete-case analysis was used for regression models. Because operation time was unavailable in 30 patients, regression models including operation time were fitted using 463 complete cases. Two-sided *p* values < 0.05 were considered statistically significant. The extent of missing data is summarized in Supplementary Table S1. Statistical analyses were performed using R software, version 4.6.0 (R Foundation for Statistical Computing, Vienna, Austria) [10].

## 3. Results

### 3.1. Study Cohort and Oxycodone Consumption

**Figure 1:**
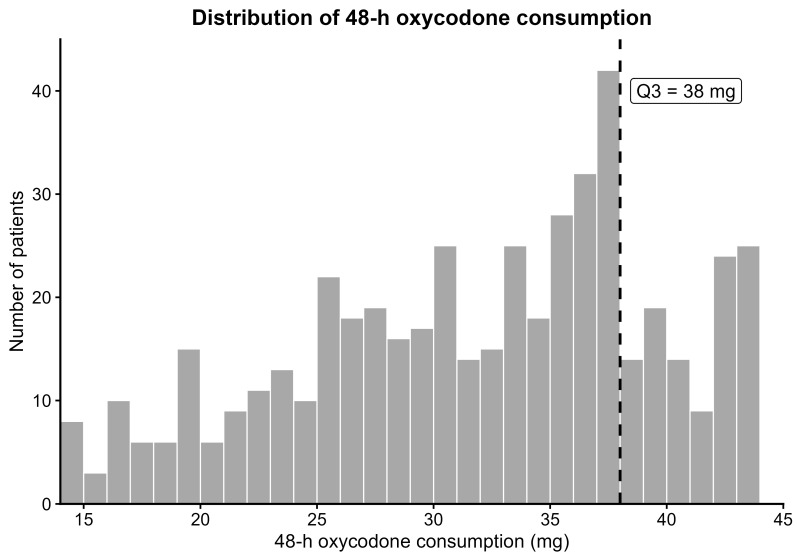
Distribution of 48 h oxycodone consumption. The dashed vertical line indicates the third quartile (Q3 = 38 mg), which was used to define the high-consumption group (>38 mg).

The final dataset included 493 patients who underwent gastrointestinal surgery and received oxycodone-based PCIA. The median cumulative 48 h oxycodone consumption was 34 mg (IQR, 27–38 mg). The third quartile of 48 h oxycodone consumption was 38 mg; therefore, high 48 h oxycodone consumption was defined as a dose >38 mg. Accordingly, 105 patients (21.3%) were classified into the high-consumption group and 388 patients (78.7%) into the non-high-consumption group. The distribution of 48 h oxycodone consumption is shown in Figure 1. Missing data were limited and are summarized in Supplementary Table S1.

### 3.2. Patient Characteristics According to High 48-Hour Oxycodone Consumption

Baseline and surgical characteristics according to high 48 h oxycodone consumption are shown in Table 1. Patients in the high-consumption group were younger than those in the non-high-consumption group (57 [50, 65] vs. 63 [53, 71] years; *p* = 0.002). Sex, height, body weight, BMI, ASA physical status, smoking history, alcohol use, hypertension, diabetes mellitus, and disease nature did not differ significantly between groups.

**Figure 2:**
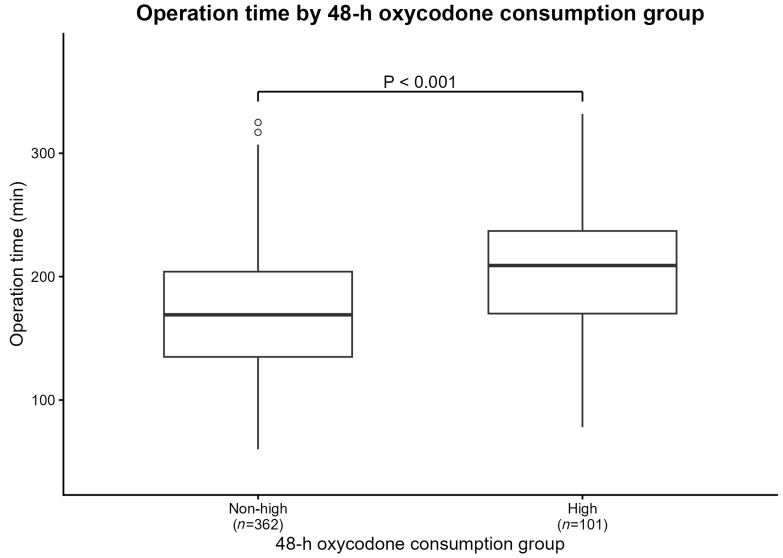
Operation time distribution according to high 48 h oxycodone consumption group. Boxes represent the interquartile range, horizontal lines indicate medians, whiskers indicate the non-outlier range, and circles indicate individual outliers defined as values outside 1.5 times the interquartile range. The analysis included patients with available operation time data (non-high group, *n* = 362; high-consumption group, *n* = 101). Operation time was significantly longer in the high-consumption group than in the non-high-consumption group (Mann–Whitney U test, *p* < 0.001).

**Figure 3:**
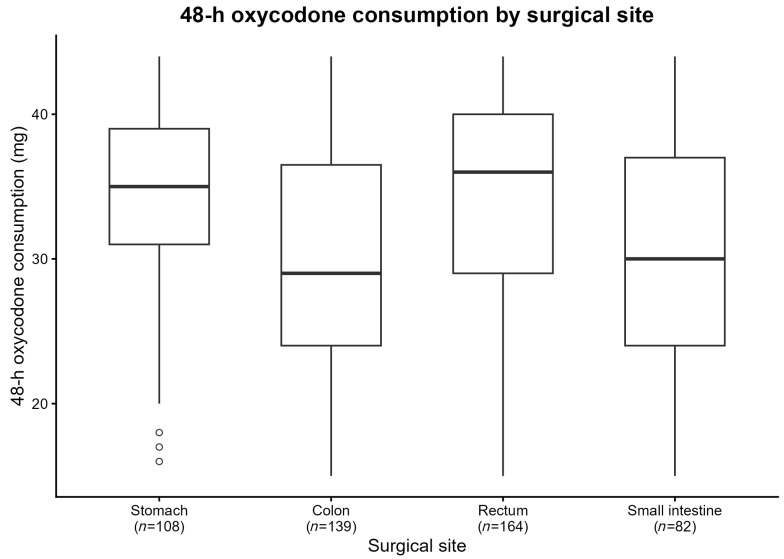
Forty-eight-hour oxycodone consumption according to surgical site. Boxes represent the interquartile range, horizontal lines indicate medians, whiskers indicate the non-outlier range, and circles indicate individual outliers defined as values outside 1.5 times the interquartile range. Surgical site groups were stomach (*n* = 108), colon (*n* = 139), rectum (*n* = 164), and small intestine (*n* = 82). Differences across surgical sites were assessed using the Kruskal–Wallis test (*p* < 0.001).

Surgical characteristics differed between groups. Surgical site distribution was significantly different (*p* < 0.001), with a higher proportion of rectal surgery in the high-consumption group than in the non-high-consumption group (46.7% vs. 29.6%). Surgical approach also differed between groups (*p* = 0.005), with open surgery and converted-to-open surgery being more frequent in the high-consumption group. Radical resection was more common in the high-consumption group than in the non-high-consumption group (71.4% vs. 42.8%; *p* < 0.001). Operation time was longer in the high-consumption group (209 [170, 237] vs. 169 [135, 204] min; *p* < 0.001), and intraoperative blood loss was greater (28 [10, 70] vs. 19 [7, 50] mL; *p* = 0.021). The distribution of operation time according to high-consumption status is shown in Figure 2, and 48 h oxycodone consumption according to surgical site is shown in Figure 3.

Among available perioperative analgesia-related variables, incisional local infiltration was less frequent in the high-consumption group than in the non-high-consumption group (32.4% vs. 45.6%; *p* = 0.020). Regional block and routine postoperative non-opioid analgesic use did not differ significantly between groups.

### 3.3. PCIA Consumption, Pain Scores, and Postoperative Outcomes

Postoperative analgesic outcomes are summarized in Table 2. The high-consumption group had higher 24 h oxycodone consumption (20 [19, 22] vs. 15 [12, 18] mg; *p* < 0.001), higher 48 h oxycodone consumption (42 [40, 43] vs. 31 [26, 36] mg; *p* < 0.001), more effective PCIA demands within 48 h, and more total PCIA demands within 48 h than the non-high-consumption group.

Pain scores were also higher in the high-consumption group. Both resting and movement-related NRS scores on POD1 and POD2 were significantly higher in the high-consumption group than in the non-high-consumption group (all *p* < 0.001). Rescue analgesia within 48 h was more frequent in the high-consumption group (43.8% vs. 20.6%; *p* < 0.001), and the number of rescue analgesic interventions was also higher (*p* < 0.001). Early PCIA pump discontinuation was less frequent in the high-consumption group (2.9% vs. 21.4%; *p* < 0.001). PCIA parameter adjustment, PONV within 48 h, drowsiness or sedation, pruritus, urinary retention, and time to first flatus did not differ significantly between groups. Postoperative length of stay was longer in the high-consumption group (9 [7, 11] vs. 7 [6, 9] days; *p* < 0.001).

### 3.4. Correlation Analysis

Spearman correlation analysis results are presented in Table 3. Cumulative 48 h oxycodone consumption was negatively correlated with age (ρ = −0.229; *p* < 0.001) and positively correlated with operation time (ρ = 0.389; *p* < 0.001) and intraoperative blood loss (ρ = 0.218; *p* < 0.001). BMI was not significantly correlated with 48 h oxycodone consumption (ρ = −0.038; *p* = 0.403).

Among postoperative pain variables, the strongest correlations with 48 h oxycodone consumption were observed for NRS scores on movement on POD1 (ρ = 0.620; *p* < 0.001) and POD2 (ρ = 0.611; *p* < 0.001). Resting NRS scores on POD1 (ρ = 0.436; *p* < 0.001) and POD2 (ρ = 0.362; *p* < 0.001) were also positively correlated with 48 h oxycodone consumption. Weak but statistically significant positive correlations were observed for the number of rescue analgesic interventions (ρ = 0.207; *p* < 0.001), time to first flatus (ρ = 0.114; *p* = 0.014), and postoperative length of stay (ρ = 0.177; *p* < 0.001).

### 3.5. Primary Continuous-Outcome Multivariable Analysis

The primary multivariable analysis evaluated cumulative 48 h oxycodone consumption as a continuous outcome using complete-case analysis of 463 patients (Table 4). In the prespecified clinical model, older age was independently associated with lower 48 h oxycodone consumption. Each 10-year increase in age was associated with a 1.03 mg lower 48 h oxycodone dose (β = −1.03; 95% CI, −1.45 to −0.61; *p* < 0.001).

Procedure-related factors were independently associated with higher 48 h oxycodone consumption. Compared with colon surgery, gastric surgery (β = 2.57 mg; 95% CI, 0.86 to 4.28; *p* = 0.003) and rectal surgery (β = 3.27 mg; 95% CI, 1.71 to 4.82; *p* < 0.001) were associated with higher 48 h oxycodone consumption. Compared with laparoscopic surgery, open surgery (β = 4.70 mg; 95% CI, 3.44 to 5.96; *p* < 0.001) and converted-to-open surgery (β = 1.94 mg; 95% CI, 0.08 to 3.80; *p* = 0.041) were associated with higher consumption. Radical resection was associated with a 3.63 mg higher 48 h oxycodone dose compared with local resection (95% CI, 2.34 to 4.92; *p* < 0.001). Each 60 min increase in operation time was associated with a 3.23 mg higher 48 h oxycodone dose (95% CI, 2.58 to 3.88; *p* < 0.001). Sex, BMI, ASA physical status, and small-intestine surgery were not independently associated with 48 h oxycodone consumption. The model R^2^ was 0.369.

In the exploratory analgesia-adjusted continuous-outcome model, the main associations remained largely unchanged. Older age remained associated with lower 48 h oxycodone consumption (β = −1.05 mg per 10-year increase; 95% CI, −1.47 to −0.63; *p* < 0.001), whereas gastric surgery, rectal surgery, open surgery, radical resection, and longer operation time remained associated with higher consumption. Converted-to-open surgery showed a positive association with 48 h oxycodone consumption, but the 95% CI crossed zero after adjustment for available analgesia-related variables (β = 1.69 mg; 95% CI, −0.12 to 3.51; *p* = 0.067).

**Figure 4:**
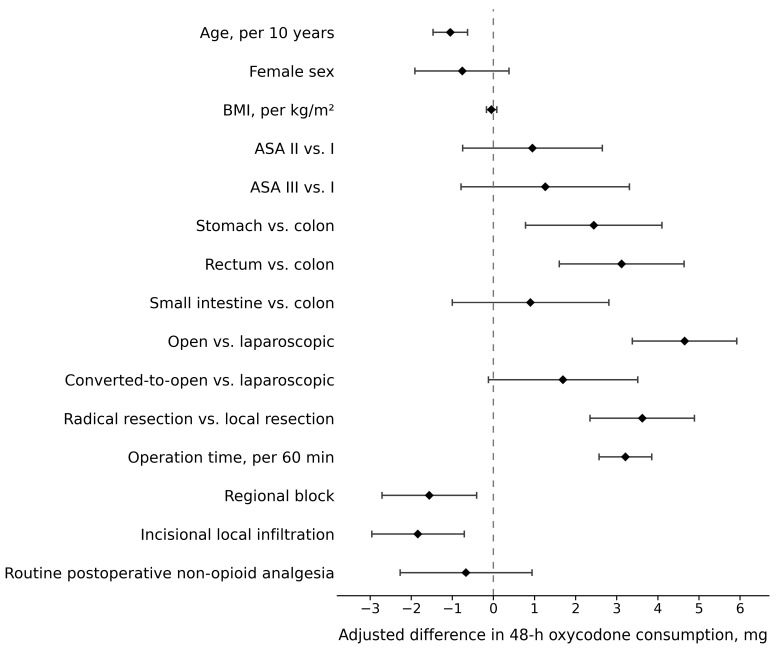
Forest plot of the analgesia-adjusted linear regression model for 48 h postoperative oxycodone consumption. Regression coefficients represent adjusted differences in cumulative 48 h oxycodone consumption in milligrams. Horizontal lines indicate 95% confidence intervals. The model included age, sex, BMI, ASA physical status, surgical site, surgical approach, extent of resection, operation time, regional block, incisional local infiltration, and routine postoperative non-opioid analgesic use. ASA, American Society of Anesthesiologists; BMI, body mass index; CI, confidence interval.

Among the added analgesia-related variables, regional block was associated with lower 48 h oxycodone consumption (β = −1.56 mg; 95% CI, −2.71 to −0.41; *p* = 0.008), and incisional local infiltration was also associated with lower consumption (β = −1.84 mg; 95% CI, −2.96 to −0.71; *p* = 0.001). Routine postoperative non-opioid analgesic use was not independently associated with 48 h oxycodone consumption (β = −0.67 mg; 95% CI, −2.27 to 0.94; *p* = 0.416). The R^2^ of the analgesia-adjusted model was 0.394. The estimates from the analgesia-adjusted continuous-outcome model are shown in Figure 4.

### 3.6. Exploratory High-Consumption Logistic Analysis

Univariable logistic regression results are provided in Supplementary Table S2. The exploratory multivariable logistic regression model included the same prespecified clinical covariates as the primary continuous-outcome model and used complete-case analysis of 463 patients (Table 5). In this model, each 10-year increase in age was associated with lower odds of high consumption (aOR, 0.78; 95% CI, 0.64–0.94; *p* = 0.010). Compared with colon surgery, gastric surgery (aOR, 2.15; 95% CI, 1.003–4.60; *p* = 0.049) and rectal surgery (aOR, 2.74; 95% CI, 1.35–5.55; *p* = 0.005) were associated with higher odds of high consumption. Open surgery (aOR, 2.47; 95% CI, 1.35–4.52; *p* = 0.003), converted-to-open surgery (aOR, 2.46; 95% CI, 1.14–5.32; *p* = 0.022), radical resection (aOR, 2.71; 95% CI, 1.55–4.73; *p* < 0.001), and longer operation time (aOR per 60 min increase, 2.46; 95% CI, 1.80–3.35; *p* < 0.001) were also independently associated with high consumption. The model AUC was 0.797 (95% CI, 0.748 to 0.845).

In the exploratory analgesia-adjusted logistic model, the main findings were broadly consistent. Older age remained associated with lower odds of high consumption (aOR per 10-year increase, 0.77; 95% CI, 0.63–0.93; *p* = 0.008). Rectal surgery (aOR, 2.66; 95% CI, 1.30–5.43; *p* = 0.007), open surgery (aOR, 2.46; 95% CI, 1.32–4.56; *p* = 0.004), converted-to-open surgery (aOR, 2.37; 95% CI, 1.09–5.20; *p* = 0.030), radical resection (aOR, 2.80; 95% CI, 1.59–4.93; *p* < 0.001), and longer operation time (aOR per 60 min increase, 2.56; 95% CI, 1.86–3.53; *p* < 0.001) remained associated with higher odds of high consumption. The association for gastric surgery was attenuated and did not reach conventional statistical significance after adjustment for available analgesia-related variables (aOR, 2.16; 95% CI, 0.998–4.65; *p* = 0.051). Incisional local infiltration was associated with lower odds of high consumption (aOR, 0.47; 95% CI, 0.28–0.79; *p* = 0.005), whereas regional block and routine postoperative non-opioid analgesic use were not independently associated with high consumption. The analgesia-adjusted logistic model had an AUC of 0.811 (95% CI, 0.767–0.853).

**Figure 5:**
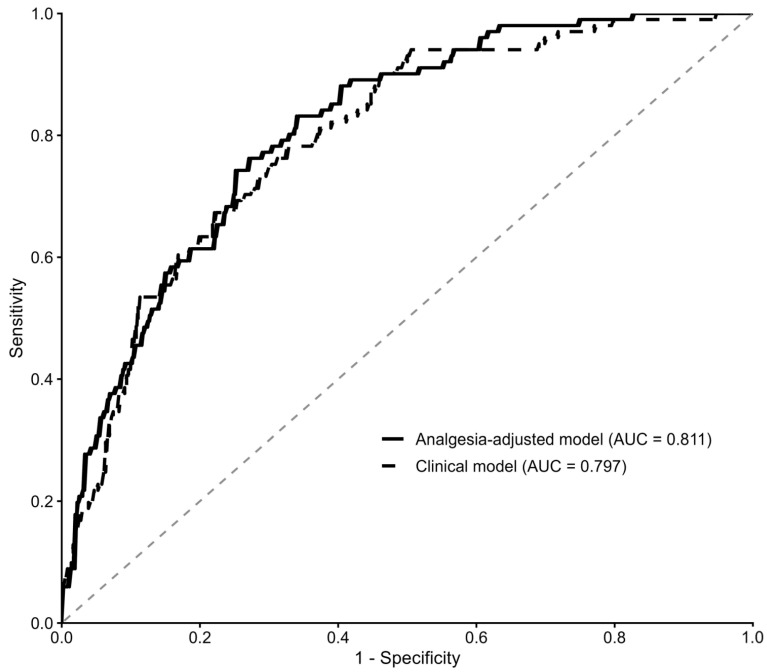
Receiver operating characteristic curves of the exploratory logistic regression models for high 48 h postoperative oxycodone consumption. High consumption was defined as a cumulative oxycodone dose >38 mg within 48 h after surgery. The clinical model included age, sex, BMI, ASA physical status, surgical site, surgical approach, extent of resection, and operation time. The analgesia-adjusted model additionally included regional block, incisional local infiltration, and routine postoperative non-opioid analgesic use. AUC values are displayed in the legend; 95% CIs are provided in Table 5. The solid black curve represents the analgesia-adjusted model, the dashed black curve represents the clinical model, and the light gray diagonal dashed line represents the no-discrimination reference (AUC = 0.5). These models were used for exploratory assessment of factors associated with relatively high oxycodone requirement and were not intended to develop or validate a clinical prediction model. ASA, American Society of Anesthesiologists; AUC, area under the receiver operating characteristic curve; BMI, body mass index; CI, confidence interval.

These exploratory logistic models were used to evaluate factors associated with relatively high oxycodone requirement within this cohort and were not intended to develop or validate a clinical prediction model. The receiver operating characteristic curves for the two exploratory logistic models are shown in Figure 5.

## 4. Discussion

In this single-center retrospective cohort study of 493 patients receiving oxycodone-based PCIA after gastrointestinal surgery, younger age and procedure-related characteristics were associated with higher 48 h oxycodone requirement. The main associations remained broadly stable after adjustment for available analgesia-related variables. Regional block and incisional local infiltration were associated with lower consumption, whereas BMI, ASA physical status, and routine postoperative non-opioid analgesic use were not independently associated. These findings should be interpreted as observational associations rather than causal effects.

The persistence of operation time, surgical approach, extent of resection, and surgical site in the adjusted models may reflect their relatively direct relationship with postoperative nociceptive burden. Prolonged operations, open procedures, and radical resections capture tissue injury, visceral manipulation, and procedural complexity, while gastric and rectal procedures may involve distinct upper abdominal or pelvic pain mechanisms. The association between longer operation time and higher opioid requirement is consistent with previous PCIA cohorts [3,8], and the procedure-specific findings support tailoring postoperative analgesic planning to surgical invasiveness and site rather than relying on a single general regimen [11,12].

Age was inversely associated with oxycodone consumption, consistent with previous observations that postoperative opioid requirements often decrease with age [3,8,13]. Potential explanations include age-related differences in pain perception, opioid pharmacokinetics and pharmacodynamics, activity, PCIA use, and clinician or patient caution [13,14]. Lower opioid consumption in older adults should not be assumed to represent better pain control, because this population may remain vulnerable to undertreatment and opioid-related adverse effects and therefore requires careful titration and reassessment [15,16].

In contrast, the absence of independent associations for BMI, ASA physical status, and postoperative non-opioid analgesic use may partly reflect limited discriminatory information. BMI values were concentrated within a relatively narrow range, 84.6% of patients had ASA class I or II, and postoperative non-opioid analgesia was documented in 85.2% of the cohort. Moreover, the non-opioid analgesia variable was binary and did not consistently capture agent, dose, timing, or frequency. These features may have reduced the ability to identify independent associations.

Although regional block and incisional local infiltration were statistically associated with lower 48 h oxycodone consumption, the adjusted absolute reductions were modest: 1.56 mg and 1.84 mg, respectively, or approximately 5% of the cohort median consumption of 34 mg. Because no minimal clinically important difference for postoperative oxycodone consumption was prespecified, statistical significance should not be assumed to indicate a clinically meaningful opioid-sparing effect. The findings are directionally consistent with multimodal analgesic principles [17,18,19,20], but their clinical relevance should be evaluated prospectively together with pain intensity, opioid-related adverse effects, functional recovery, and patient-reported benefit.

These analgesic techniques were not protocolized or randomly assigned and may have been selected according to surgical site, procedural complexity, clinician preference, anticipated pain intensity, or patient-specific considerations. Confounding by indication therefore cannot be excluded, and the observed associations should be considered hypothesis-generating. In addition, detailed cumulative intraoperative opioid exposure was not available for the full cohort. Remifentanil dosing and supplemental opioid administration were individualized according to surgical stimulation, hemodynamic responses, and clinical judgment; their omission from the models remains an important source of residual confounding.

Routine postoperative non-opioid analgesic use was not independently associated with oxycodone requirement. This should not be interpreted as evidence that non-opioid analgesia was ineffective. Flurbiprofen axetil was the principal non-opioid analgesic used, but the retrospective database reliably captured only whether postoperative non-opioid treatment was documented; patient-level agent, dose, timing, and frequency were not consistently retrievable. The binary exposure may therefore have inadequately represented the intensity and quality of multimodal analgesia.

Postoperative pain scores and rescue analgesia were correlated with oxycodone consumption, as expected. These variables occurred after PCIA initiation and were not included in the main models because they may represent downstream consequences of inadequate analgesia or opioid exposure rather than baseline predictors. They remain clinically useful as dynamic indicators for acute pain service reassessment.

The exploratory logistic models showed moderate discrimination, with AUCs of 0.797 and 0.811. The small increase after adding available analgesia-related variables should not be overinterpreted. These models were intended to explore factors associated with relatively high consumption within this cohort, not to establish a clinical prediction tool; calibration and external validation would be required before any risk score could be considered.

Clinically, the findings suggest that routinely available information—particularly age, surgical site, approach, extent of resection, and operation time—may help identify patients who warrant earlier pain reassessment, closer postoperative monitoring, reinforced PCIA education, and individualized multimodal analgesic planning. This approach may be more useful than uniform escalation of opioid therapy.

Prospective multicenter studies with standardized capture of intraoperative opioid exposure, regional and local techniques, non-opioid drug regimens, psychological factors, and patient-centered outcomes are needed to determine whether risk-informed care improves analgesia while limiting adverse effects and unnecessary opioid exposure.

### Limitations

This study has several limitations. First, its single-center retrospective design precludes causal inference and may introduce selection, information, and residual confounding. The findings reflect one institution’s surgical case mix, PCIA regimen, and acute pain service workflow and may not generalize to other settings.

Second, the sample size was limited, with 105 high-consumption events in the full cohort and 101 in the complete-case dataset. The primary analysis therefore retained oxycodone consumption as a continuous outcome, while the dichotomized logistic analyses were exploratory and should not be interpreted as validated prediction models.

Third, detailed anesthesia and analgesia exposures were incompletely captured. Cumulative intraoperative remifentanil administration and supplemental opioid doses were not consistently retrievable and could not be included in the models. Flurbiprofen axetil was the principal non-opioid analgesic, but the specific agent, dose, timing, and frequency were not consistently available for all patients; postoperative non-opioid analgesia was therefore analyzed only as a binary variable. Local anesthetic dose and other adjunctive analgesic exposures were also incompletely recorded, leaving residual confounding related to anesthetic technique and multimodal analgesia.

Fourth, regional block, incisional local infiltration, and non-opioid analgesia were not protocolized or randomly assigned. Their use may have reflected procedural complexity, clinician preference, anticipated pain intensity, or patient-specific factors; consequently, their associations should not be interpreted as causal evidence of treatment efficacy.

Fifth, the dataset did not include preoperative anxiety, depressive symptoms, pain catastrophizing, previous pain experiences, expectations regarding pain control, or other patient-reported psychological factors. These variables may influence pain perception, PCIA use, and analgesic consumption and represent additional sources of residual confounding [21,22].

Sixth, 48 h oxycodone consumption was derived from pump and analgesia records and may have been influenced by patient understanding, nursing instruction, parameter adjustment, early pump discontinuation, and rescue analgesia. These postoperative process variables were described but were not included as baseline predictors.

Seventh, the >38 mg high-consumption threshold was cohort-specific and should not be considered a universal clinical cutoff. Some variables also had missing values, and complete-case regression reduced the analysis sample from 493 to 463 patients; bias remains possible if missingness was not random.

Finally, the study did not assess persistent postoperative pain, opioid use after discharge, functional recovery, readmission, or patient-reported quality of recovery. Future multicenter prospective studies with standardized medication exposure and longer follow-up are needed to confirm the findings and assess their clinical utility [23,24,25].

## 5. Conclusions

In patients receiving oxycodone-based PCIA after gastrointestinal surgery, younger age and procedure-related characteristics, including surgical site, surgical approach, extent of resection, and operation time, were associated with higher 48 h postoperative oxycodone requirement. These routinely available perioperative factors may help acute pain service teams prioritize patients for earlier pain reassessment, closer postoperative monitoring, reinforced PCIA education, and individualized multimodal analgesic planning. Regional block and incisional local infiltration were associated with modestly lower oxycodone consumption, but their clinical importance remains uncertain and these observational associations should not be interpreted as causal treatment effects. Prospective multicenter validation is needed before formal risk-stratified analgesic protocols can be recommended.

## Figures and Tables

**Table 1:** Baseline and surgical characteristics according to high 48 h oxycodone consumption.

Variable	Overall (*n* = 493)	Non-High Consumption (*n* = 388)	High Consumption (*n* = 105)	*p* Value
Age, years	62 (52, 71)	63 (53, 71)	57 (50, 65)	0.002
Sex				1.000
Male	251 (50.9)	198 (51.0)	53 (50.5)	
Female	242 (49.1)	190 (49.0)	52 (49.5)	
Height, cm	165 (160, 170)	165 (160, 170)	165 (161, 170)	0.999
Body weight, kg	60 (53, 67)	60 (54, 67)	58 (52, 65)	0.086
BMI, kg/m^2^	22.1 (19.4, 24.6)	22.3 (19.4, 24.9)	21.5 (19.3, 23.6)	0.121
ASA physical status				0.726
I	80 (16.2)	64 (16.5)	16 (15.2)	
II	337 (68.4)	262 (67.5)	75 (71.4)	
III	76 (15.4)	62 (16.0)	14 (13.3)	
Smoking history				0.428
Yes	174 (35.3)	133 (34.3)	41 (39.0)	
No	319 (64.7)	255 (65.7)	64 (61.0)	
Alcohol use				0.895
Yes	122 (24.7)	95 (24.5)	27 (25.7)	
No	371 (75.3)	293 (75.5)	78 (74.3)	
Hypertension				0.789
Yes	208 (42.2)	162 (41.8)	46 (43.8)	
No	285 (57.8)	226 (58.2)	59 (56.2)	
Diabetes mellitus				0.828
Yes	88 (17.8)	68 (17.5)	20 (19.0)	
No	405 (82.2)	320 (82.5)	85 (81.0)	
Disease nature				0.673
Malignant	294 (59.6)	229 (59.0)	65 (61.9)	
Benign	199 (40.4)	159 (41.0)	40 (38.1)	
Surgical site				<0.001
Stomach	108 (21.9)	79 (20.4)	29 (27.6)	
Colon	139 (28.2)	123 (31.7)	16 (15.2)	
Rectum	164 (33.3)	115 (29.6)	49 (46.7)	
Small intestine	82 (16.6)	71 (18.3)	11 (10.5)	
Surgical approach				0.005
Laparoscopic	345 (70.0)	285 (73.5)	60 (57.1)	
Open	101 (20.5)	70 (18.0)	31 (29.5)	
Converted-to-open	47 (9.5)	33 (8.5)	14 (13.3)	
Extent of resection				<0.001
Radical resection	241 (48.9)	166 (42.8)	75 (71.4)	
Local resection	252 (51.1)	222 (57.2)	30 (28.6)	
Stoma creation				0.311
Yes	89 (18.1)	66 (17.0)	23 (21.9)	
No	404 (81.9)	322 (83.0)	82 (78.1)	
Operation time, min	177 (143, 217)	169 (135, 204)	209 (170, 237)	<0.001
Blood loss, mL	20 (8, 53)	19 (7, 50)	28 (10, 70)	0.021
Transfusion				0.105
Yes	22 (4.5)	14 (3.6)	8 (7.6)	
No	471 (95.5)	374 (96.4)	97 (92.4)	
Regional block				0.097
Yes	277 (56.2)	226 (58.2)	51 (48.6)	
No	216 (43.8)	162 (41.8)	54 (51.4)	
Incisional local infiltration				0.020
Yes	211 (42.8)	177 (45.6)	34 (32.4)	
No	282 (57.2)	211 (54.4)	71 (67.6)	
Routine postoperative non-opioid analgesia				0.361
Yes	420 (85.2)	334 (86.1)	86 (81.9)	
No	73 (14.8)	54 (13.9)	19 (18.1)	

Note: Data are presented as median (interquartile range) or *n* (%). Continuous variables were summarized using available data; denominators may vary because of missing values. *p* values compare the non-high-consumption and high-consumption groups using the Mann–Whitney U test, chi-square test, or Fisher’s exact test, as appropriate. The between-group comparisons were descriptive and exploratory; no adjustment for multiple comparisons was applied. ASA, American Society of Anesthesiologists; BMI, body mass index.

**Table 2:** PCIA consumption, pain scores, and postoperative outcomes.

Variable	Overall (*n* = 493)	Non-High Consumption (*n* = 388)	High Consumption (*n* = 105)	*p* Value
24 h oxycodone consumption, mg	16 (13, 19)	15 (12, 18)	20 (19, 22)	<0.001
48 h oxycodone consumption, mg	34 (27, 38)	31 (26, 36)	42 (40, 43)	<0.001
Effective PCIA demands within 48 h	22 (15, 26)	19 (14, 24)	30 (28, 31)	<0.001
Total PCIA demands within 48 h	23 (17, 28)	21 (15, 25)	32 (30, 33)	<0.001
PCIA parameter adjustment	52 (10.5)	39 (10.1)	13 (12.4)	0.610
Early PCIA pump discontinuation	86 (17.4)	83 (21.4)	3 (2.9)	<0.001
POD1 NRS at rest	2 (1, 3)	2 (1, 3)	3 (2, 4)	<0.001
POD1 NRS on movement	4 (3, 6)	4 (3, 5)	6 (5, 7)	<0.001
POD2 NRS at rest	1 (0, 2)	1 (0, 2)	2 (1, 3)	<0.001
POD2 NRS on movement	3 (2, 4)	3 (1, 4)	4 (3, 5)	<0.001
PONV within 48 h	128 (26.0)	97 (25.0)	31 (29.5)	0.416
Rescue analgesia within 48 h	126 (25.6)	80 (20.6)	46 (43.8)	<0.001
Number of rescue analgesic interventions	0 (0, 1)	0 (0, 0)	0 (0, 1)	<0.001
Dizziness	71 (14.4)	50 (12.9)	21 (20.0)	0.092
Drowsiness/sedation	36 (7.3)	26 (6.7)	10 (9.5)	0.438
Pruritus	29 (5.9)	23 (5.9)	6 (5.7)	1.000
Urinary retention	23 (4.7)	17 (4.4)	6 (5.7)	0.602
Time to first flatus, d	3 (2, 4)	3 (2, 3)	3 (2, 4)	0.156
Postoperative length of stay, d	8 (6, 10)	7 (6, 9)	9 (7, 11)	<0.001

Note: Data are presented as median (interquartile range) or *n* (%). Continuous variables were summarized using available data; denominators may vary because of missing values. NRS was assessed at rest and on movement. PCIA, patient-controlled intravenous analgesia; POD, postoperative day; NRS, numerical rating scale; PONV, postoperative nausea and vomiting.

**Table 3:** Spearman correlation analysis with 48 h oxycodone consumption.

Variable	*n*	Spearman ρ	*p* Value
Age, years	493	−0.229	<0.001
BMI, kg/m^2^	493	−0.038	0.403
Operation time, min	463	0.389	<0.001
Blood loss, mL	466	0.218	<0.001
POD1 NRS at rest	470	0.436	<0.001
POD1 NRS on movement	468	0.620	<0.001
POD2 NRS at rest	468	0.362	<0.001
POD2 NRS on movement	468	0.611	<0.001
Number of rescue analgesic interventions	493	0.207	<0.001
Time to first flatus, d	464	0.114	0.014
Postoperative length of stay, d	464	0.177	<0.001

Note: Positive coefficients indicate higher 48 h oxycodone consumption with increasing values of the variable; negative coefficients indicate lower consumption. Postoperative pain scores, rescue analgesia, time to first flatus, and postoperative length of stay were analyzed descriptively and were not included in the main multivariable models because they may occur after PCIA initiation or represent downstream clinical outcomes. BMI, body mass index; NRS, numerical rating scale; POD, postoperative day.

**Table 4:** Primary continuous-outcome linear regression models for 48 h postoperative oxycodone consumption.

Variable	Clinical Model β (95% CI)	*p* Value	Analgesia-Adjusted Model β (95% CI)	*p* Value
Age, per 10 years	−1.03 (−1.45 to −0.61)	<0.001	−1.05 (−1.47 to −0.63)	<0.001
Female sex	−0.54 (−1.70 to 0.62)	0.359	−0.76 (−1.91 to 0.38)	0.193
BMI, per kg/m^2^	−0.06 (−0.18 to 0.07)	0.359	−0.05 (−0.17 to 0.08)	0.481
ASA II vs. I	0.93 (−0.73 to 2.59)	0.272	0.95 (−0.75 to 2.65)	0.274
ASA III vs. I	1.05 (−1.03 to 3.12)	0.324	1.26 (−0.79 to 3.31)	0.229
Stomach vs. colon	2.57 (0.86 to 4.28)	0.003	2.44 (0.78 to 4.10)	0.004
Rectum vs. colon	3.27 (1.71 to 4.82)	<0.001	3.12 (1.60 to 4.64)	<0.001
Small intestine vs. colon	0.97 (−0.94 to 2.89)	0.320	0.90 (−1.00 to 2.81)	0.351
Open vs. laparoscopic	4.70 (3.44 to 5.96)	<0.001	4.65 (3.38 to 5.92)	<0.001
Converted-to-open vs. laparoscopic	1.94 (0.08 to 3.80)	0.041	1.69 (−0.12 to 3.51)	0.067
Radical resection vs. local resection	3.63 (2.34 to 4.92)	<0.001	3.62 (2.35 to 4.89)	<0.001
Operation time, per 60 min	3.23 (2.58 to 3.88)	<0.001	3.21 (2.57 to 3.85)	<0.001
Regional block	—	—	−1.56 (−2.71 to −0.41)	0.008
Incisional local infiltration	—	—	−1.84 (−2.96 to −0.71)	0.001
Routine postoperative non-opioid analgesia	—	—	−0.67 (−2.27 to 0.94)	0.416

Note: The outcome was cumulative oxycodone consumption within 48 h after surgery, analyzed as a continuous variable in milligrams. Both models used complete-case analysis (*n* = 463). Regression coefficients are adjusted differences in 48 h oxycodone consumption. The clinical model included age, sex, BMI, ASA physical status, surgical site, surgical approach, extent of resection, and operation time. The analgesia-adjusted model additionally included regional block, incisional local infiltration, and routine postoperative non-opioid analgesic use. Linear regression models were fitted with heteroscedasticity-robust standard errors. Model R^2^ was 0.369 for the clinical model and 0.394 for the analgesia-adjusted model. Reference categories were male sex, ASA I, colon surgery, laparoscopic surgery, local resection, and absence of each analgesia technique. ASA, American Society of Anesthesiologists; BMI, body mass index; CI, confidence interval.

**Table 5:** Exploratory logistic regression models for high 48 h postoperative oxycodone consumption.

Variable	Clinical Model aOR (95% CI)	*p* Value	Analgesia-Adjusted Model aOR (95% CI)	*p* Value
Age, per 10 years	0.78 (0.64 to 0.94)	0.010	0.77 (0.63 to 0.93)	0.008
Female sex	1.00 (0.61 to 1.66)	0.985	0.94 (0.56 to 1.57)	0.813
BMI, per kg/m^2^	0.97 (0.91 to 1.02)	0.249	0.96 (0.91 to 1.03)	0.246
ASA II vs. I	1.22 (0.60 to 2.49)	0.574	1.25 (0.61 to 2.55)	0.549
ASA III vs. I	0.96 (0.38 to 2.40)	0.929	1.00 (0.39 to 2.54)	0.998
Stomach vs. colon	2.15 (1.003 to 4.60)	0.049	2.16 (0.998 to 4.65)	0.051
Rectum vs. colon	2.74 (1.35 to 5.55)	0.005	2.66 (1.30 to 5.43)	0.007
Small intestine vs. colon	1.23 (0.48 to 3.14)	0.660	1.20 (0.46 to 3.13)	0.706
Open vs. laparoscopic	2.47 (1.35 to 4.52)	0.003	2.46 (1.32 to 4.56)	0.004
Converted-to-open vs. laparoscopic	2.46 (1.14 to 5.32)	0.022	2.37 (1.09 to 5.20)	0.030
Radical resection vs. local resection	2.71 (1.55 to 4.73)	< 0.001	2.80 (1.59 to 4.93)	< 0.001
Operation time, per 60 min	2.46 (1.80 to 3.35)	< 0.001	2.56 (1.86 to 3.53)	< 0.001
Regional block	—	—	0.68 (0.41 to 1.13)	0.140
Incisional local infiltration	—	—	0.47 (0.28 to 0.79)	0.005
Routine postoperative non-opioid analgesia	—	—	1.03 (0.51 to 2.08)	0.939

Note: High 48 h oxycodone consumption was defined as a cumulative dose > 38 mg. Both logistic regression models used complete-case analysis (*n* = 463), including 101 high-consumption events. The clinical model included age, sex, BMI, ASA physical status, surgical site, surgical approach, extent of resection, and operation time. The analgesia-adjusted model additionally included regional block, incisional local infiltration, and routine postoperative non-opioid analgesic use. The AUC was 0.797 (95% CI, 0.748 to 0.845) for the clinical model and 0.811 (95% CI, 0.767 to 0.853) for the analgesia-adjusted model. These models were used for exploratory assessment of factors associated with relatively high oxycodone requirement and were not intended to develop or validate a clinical prediction model. Reference categories were male sex, ASA I, colon surgery, laparoscopic surgery, local resection, and absence of each analgesia technique. aOR, adjusted odds ratio; ASA, American Society of Anesthesiologists; AUC, area under the receiver operating characteristic curve; BMI, body mass index; CI, confidence interval.

## Data Availability

The de-identified datasets generated and/or analyzed during the current study are not publicly available because they contain patient-level clinical information and are subject to institutional and ethical restrictions related to patient privacy. The data may be available from the corresponding author upon reasonable request and with permission from Quanzhou First Hospital.

## Supplementary Materials

The following supporting information can be downloaded at: https://www.mdpi.com/article/10.3390/jcm15155957/s1,Table S1. Missing data summary; Table S2. Univariable logistic regression for high 48 h oxycodone consumption.

## Abbreviations

The following abbreviations are used in this manuscript:

aOR, adjusted odds ratio; APS, acute pain service; ASA, American Society of Anesthesiologists; AUC, area under the receiver operating characteristic curve; BMI, body mass index; CI, confidence interval; IQR, interquartile range; NRS, numerical rating scale; OR, odds ratio; PCIA, patient-controlled intravenous analgesia; POD, postoperative day; PONV, postoperative nausea and vomiting; Q3, third quartile.
