# Supplementary material for: Perioperative Factors Associated with 48-Hour Postoperative Oxycodone Requirement After Gastrointestinal Surgery: A Single-Center Retrospective Cohort Study
Source: J Clin Med. 2026 Jul 30;15(15):5957. doi: 10.3390/jcm15155957 (PMC13466669; doi:10.3390/jcm15155957)
Supplement: Supplementary file 1 [file jcm-15-05957-s001.zip › jcm-4445370-supplementary.pdf]

Supplementary Table S1. Missing data summary

Variable	Total *n*	Available *n*	Missing *n* (%)
Operation time	493	463	30 (6.1)
Blood loss	493	466	27 (5.5)
POD1 NRS at rest	493	470	23 (4.7)
POD1 NRS on movement	493	468	25 (5.1)
POD2 NRS at rest	493	468	25 (5.1)
POD2 NRS on movement	493	468	25 (5.1)
Time to first flatus	493	464	29 (5.9)
Postoperative length of stay	493	464	29 (5.9)

Note: Percentages were calculated using the full cohort (n = 493) as the denominator. All remaining variables used in the analyses had complete data. POD, postoperative day; NRS, numerical rating scale.

Supplementary Table S2. Univariable logistic regression for high 48 h oxycodone consumption

Variable	*n*	OR	95% CI	*p* value
Age per 10-year increase	493	0.77	0.65 to 0.91	0.002
BMI per 1-kg/m² increase	493	0.97	0.92 to 1.02	0.228
Operation time per 60-min increase	463	2.16	1.64 to 2.84	<0.001
Blood loss per 50-mL increase	466	1.18	1.00 to 1.40	0.049
Female sex	493	1.02	0.66 to 1.57	0.920
ASA II vs. I	493	1.15	0.63 to 2.10	0.661
ASA III vs. I	493	0.90	0.41 to 2.01	0.803
Malignant disease vs benign	493	1.13	0.72 to 1.76	0.593
Small intestine vs colon	493	1.19	0.52 to 2.71	0.677
Rectum vs colon	493	3.28	1.76 to 6.08	<0.001
Stomach vs colon	493	2.82	1.44 to 5.53	0.003
Open vs laparoscopic	493	2.10	1.27 to 3.49	0.004
Converted-to-open vs laparoscopic	493	2.02	1.02 to 3.99	0.045
Radical resection	493	3.34	2.09 to 5.34	<0.001
Stoma creation	493	1.37	0.80 to 2.33	0.249
Regional block	493	0.68	0.44 to 1.04	0.077
Incisional local infiltration	493	0.57	0.36 to 0.90	0.016
Routine postoperative non-opioid analgesia	493	0.73	0.41 to 1.30	0.286
PONV within 48 h	493	1.26	0.78 to 2.03	0.349
Rescue analgesia within 48 h	493	3.00	1.90 to 4.74	<0.001
Early PCIA pump discontinuation	493	0.11	0.03 to 0.35	<0.001

Note: High consumption was defined as >38 mg within 48 h. Reference categories were male sex, ASA I, benign disease, colon surgery, laparoscopic approach, local resection, no stoma creation, no regional block, no incisional infiltration, no routine postoperative non-opioid analgesia, no PONV, no rescue analgesia, and no early PCIA pump discontinuation. Postoperative process variables were included in exploratory univariable analyses but were not included in the main multivariable models because they may occur after PCIA initiation or represent downstream clinical outcomes. ASA, American Society of Anesthesiologists; BMI, body mass index; CI, confidence interval; OR, odds ratio; PCIA, patient-controlled intravenous analgesia; PONV, postoperative nausea and vomiting. **Univariable analyses were exploratory and should be interpreted descriptively; no adjustment for multiple comparisons was applied.**
